# Olfactory Dysfunction Is Associated with the Intake of Macronutrients in Korean Adults

**DOI:** 10.1371/journal.pone.0164495

**Published:** 2016-10-10

**Authors:** Il Gyu Kong, So Young Kim, Min-Su Kim, Bumjung Park, Jin-Hwan Kim, Hyo Geun Choi

**Affiliations:** 1 Department of Otorhinolaryngology-Head & Neck Surgery, Hallym University Sacred Heart Hospital, Hallym University College of Medicine, Anyang, Korea; 2 Department of Otorhinolaryngology - Head and Neck Surgery, CHA Bundang Medical Center, CHA University, Seongnam, South Korea; 3 Department of Otorhinolaryngology-Head and Neck Surgery, Korea University Ansan Hospital, Gyeonggi-do, Korea; 4 Department of Otorhinolaryngology-Head & Neck Surgery, Hallym University College of Medicine, Seoul, Korea; Inha University, REPUBLIC OF KOREA

## Abstract

**Background:**

Olfactory function can impact food selection. However, few large population-based studies have investigated this effect across different age groups. The objective of this study was to assess the association between subjective olfactory dysfunction (anosmia or hyposmia) and macronutrient intake.

**Methods:**

A total of 24,990 participants aged 20 to 98 years were evaluated based on data collected through the Korea National Health and Nutrition Examination Survey from 2008 through 2012. Olfactory dysfunction was surveyed using a self-reported questionnaire, and the nutritional status was assessed through a validated 24-hour recall method. Simple and multiple linear regression analyses with complex sampling were performed to evaluate the relationships between olfactory dysfunction and protein intake (daily protein intake/recommended protein intake [%]), carbohydrate intake (daily carbohydrate intake/total calories [%]), and fat intake (daily fat intake/total calories [%]) after adjusting for age, sex, body mass index, income, smoking history, alcohol consumption, and stress level.

**Results:**

Olfactory dysfunction was reported by 5.4% of Korean adults and was found to be associated with decreased fat consumption (estimated value [EV] of fat intake [%] = -0.57, 95% confidence interval [CI] = -1.13 to -0.13, P = 0.045). A subgroup analysis according to age and sex revealed that among young females, olfactory dysfunction was associated with reduced fat consumption (EV = -2.30, 95% CI = -4.16 to -0.43, P = 0.016) and increased carbohydrate intake (EV = 2.80, 95% CI = 0.55 to 5.05, P = 0.015), and that among middle-aged females, olfactory dysfunction was also associated with reduced fat intake (EV = -1.26, 95% CI = -2.37 to -0.16, P = 0.025). In contrast, among young males, olfactory dysfunction was associated with reduced protein intake (EV = -26.41 95% CI = -45.14 to -7.69, P = 0.006).

**Conclusion:**

Olfactory dysfunction was associated with reduced fat intake. Moreover, olfactory dysfunction exerted differential effects on eating behavior depending on age and sex.

## Introduction

Olfaction is a vital sensory function that is directly associated with food preferences and food selection [1]. Therefore, most people with olfactory dysfunction report that they feel that food is less enjoyable and less flavorful after loss of olfactory function [2–4]. Subsequently, these feelings have the potential to affect the dietary habits of people with olfactory dysfunction [5]. Some investigators have stated that olfactory dysfunction is related to decreased appetite, an alteration of dietary habits, eating less, the use more spices, and the consumption of fewer sweets [6, 7]. The loss of olfactory function is particularly important in elderly people due to the high prevalence of this condition in this population and because loss of appetite could influence malnutrition among the elderly [8, 9]. However, the results of studies on olfaction and nutrition have yielded inconsistent results [6]. Some studies have found that patients with olfactory dysfunction do not exhibit differences in terms of body mass index (BMI), nutritional intake, and eating frequency [10], even though they eat less [7]. Gopinath et al. reported that olfactory loss is related to reduced overall diet quality [11]. Some researchers have found that loss of olfaction is associated with a reduced consumption of sweets [7], whereas other authors have reported an increased intake of sweets [1].

Olfactory function is thought to directly affect food intake behavior, but few studies have addressed this issue using a large population-based study design. We evaluated the association between subjective olfactory dysfunction (anosmia or hyposmia) and macronutrient intake based on the Korea National Health and Nutrition Examination Survey (KNHANES). To the best of our knowledge, this study constitutes the largest population-based evaluation of these associations. Moreover, this study analyzed these relationships across different age groups, an analysis that has been performed in few studies.

## Materials and Methods

### Study Population and Survey Used for Data Collection

This study was approved by the Institutional Review Board of the Korea Centers for Disease Control and Prevention (2008-04EXP-01-C; 2009-01CON-03-2C; 2010-02CON-21-C; 2011-02CON-06-C; 2012-01EXP-01-2C). Written informed consent was obtained from all participants prior to the survey.

This investigation was a cross-sectional study that utilized data from the KNHANES, which included participants who were representative of the South Korean population. The survey included a health interview, a nutritional survey, and physical examinations. The applied statistical methods were based on the implemented sampling design and utilized weighted values. KNHANES data collected by the Centers for Disease Control and Prevention of Korea from 2009 to 2012 were analyzed. Each year, 192 districts were selected by a panel, and 20 households in each of these districts were further identified to perform sampling that was reflective of the entire Korean population. The surveys evaluated data from the civilian, non-institutionalized South Korean population using a stratified, multistage, clustered sampling method that was based on national census data. The sample was weighted by statisticians who performed post-stratification and accounted for the non-response rates and for extreme values. The details of the methods used to perform these procedures are provided in the KNHANES report [12].

**Fig 1:**
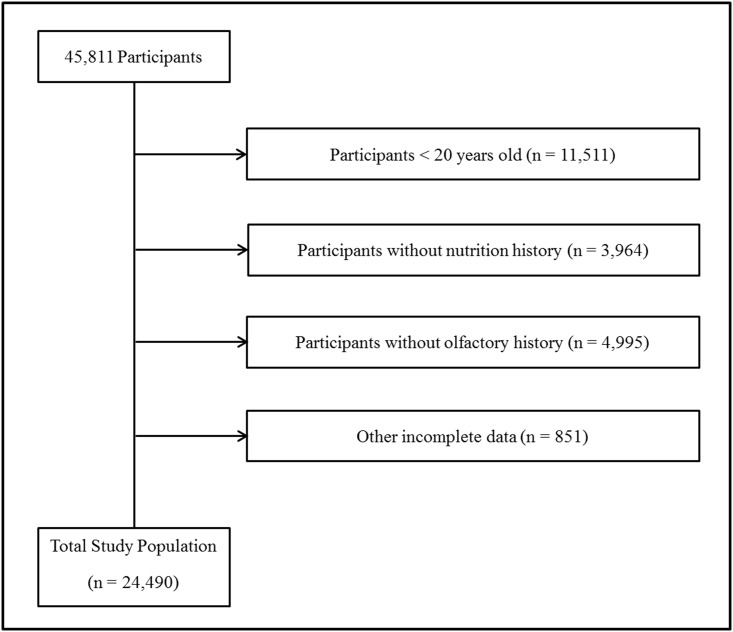
A schematic illustration of the participant selection process that was used in the present study. Out of a total of 45,811 survey respondents, 21,321 respondents (those aged less than 20 years or who lacked records for nutritional, olfactory, or other data) were excluded; as a result, 24,490 participants were included.

Among the 45,811 participants, who were 0 to 98 years of age, we excluded the following participants from this study: participants aged less than 20 years (11,511 participants), participants who did not complete the nutritional survey (3,964 participants), participants who did not respond to the questionnaire regarding their history of subjective olfactory dysfunction (4,995 participants), and participants with incomplete information regarding their BMI (kg/m^2^), income level, smoking history, alcohol consumption, and stress level (851 participants). Ultimately, 24,490 participants (9,827 males and 14,663 females) were included in this study (Fig 1).

### Data acquisition

Food intake data were surveyed by trained staff using the complete 24-hour recall method, which was validated in a previous study [13]. Responses on certain days, such as holidays and weekends, were not included. The intake of total calories (kcal/day), total protein (g/day), water (ml/day), and sodium (Na, g/day) were calculated based on the reference nutrient concentrations in foods according to the Korean Food Composition Table [14]. The intake was compared with the reference recommended dietary intake for Korean individuals [15]. Therefore, the relative intake of total calories, total protein, water, and sodium for each participant was calculated after adjusting for age and sex. For example, the relative total calorie intake (%) was calculated as the total calorie intake (cal) divided by the age- and sex-matched recommended total calorie intake (cal), and the relative total protein intake (%) was calculated as the total protein intake (g) divided by the age- and sex-matched recommended total protein intake (g). In contrast to other nutritional components, the balance between proteins, fats and carbohydrates is the most important aspect of fat and carbohydrate intake measurements [15]. Thus, the fat and carbohydrate intakes were measured relative to the total calorie intake rather than to the age- and sex-matched recommended intake [16]. As a result, the fat and carbohydrate intakes were measured using as the fat calorie intake divided by the total calorie intake (%) and the carbohydrate calorie intake divided by the total calorie intake (%), respectively.

According to the BMI classifications for the Asia-Pacific region [17], the BMI can be categorized into three groups: low BMI group, < 18.5; normal BMI group, 18.5–25.0; and high BMI group, ≥ 25.0. By dividing the household income by the square root of the number of household members, the monthly income was divided into four quartiles from top to bottom: lowest, lower middle, upper middle, and highest. The smoking history was divided into two categories: smoked fewer than five packs (100 cigarettes) in a lifetime and smoked five or more packs in a lifetime. Alcohol consumption was divided into three categories: less than once a month, one to four times a month, and more than twice a week. The participants were asked if they usually feel stress, and the stress level was divided into the following four categories: no stress, little stress, moderate stress, and severe stress. We then regrouped these categories to obtain two groups: no or some stress and moderate or severe stress.

To evaluate subjective olfactory dysfunction, the participants were asked whether they had problems with their sense of smell during the past three months. The participants who subjectively complained of anosmia or hyposmia were classified as having olfactory dysfunction.

### Statistical Analysis

The differences in mean age and nutritional factors between the participants with subjective olfactory dysfunction and the participants without subjective olfactory dysfunction (control group) were assessed via linear regression analysis with complex sampling. The significance of the differences in sex, BMI classification, income level, smoking history, alcohol consumption, and stress level between the two groups was determined using the Chi-square test with the Rao-Scott correction.

Estimated values (EVs) for the olfactory dysfunction group were calculated via simple linear regression analysis with complex sampling (unadjusted) and via multiple linear regression analysis with complex sampling and adjusting for age, sex, BMI, income, smoking history, alcohol consumption, and stress level (full adjusted model). For the subgroup analysis, the participants were divided into six groups according to age and sex: young males (20–39 y), middle-aged males (40–59 y), elderly males (60+ y), young females (20–39 y), middle-aged females (40–59 y), and elderly females (60+). Multiple linear regression analyses with complex sampling and adjusting for age, sex, BMI, income, smoking history, alcohol consumption, and stress level (fully adjusted model) was then performed for the subgroup analyses. Two-tailed analyses were conducted, and *P*-values less than 0.05 were considered to indicate significance. EVs and 95% confidence intervals (CIs) were calculated. After applying the weighted values as recommended by the KNHANES report, all results are presented as weighted values. The results were statistically analyzed using SPSS ver. 22.0 (IBM, Armonk, NY, USA).

## Results

Of the total 24,490 participants, olfactory dysfunction was reported by 5.4% (1,332 participants), and the results are reported in S1 Table according to age and sex. The mean age of the individuals with olfactory dysfunction was 54.7 years, which was older than the mean age of 45.9 years of the control group. The income level, alcohol consumption, and stress level were significantly different between the olfactory dysfunction and control groups, whereas sex, BMI, and smoking history did not present significant differences between the two groups (Table 1). Therefore, a multiple regression analysis was performed after adjustment for these nonsignificant factors.

**Table 1:** General characteristics of participants with normal olfactory function and participants with olfactory dysfunction.

	Control Group	Olfactory Dysfunction Group	P-value
Total Number			
Number	23,158	1,332	
%	96.4	5.4	
Mean Age (y)	45.9	54.7	<0.001*
Sex (%)			0.922
Male	46.4	46.5	
Female	53.6	53.5	
BMI (kg/m^2^) (%)			0.286
< 18.5	5.0	4.0	
≥ 18.5, < 25.0	63.2	62.1	
≥ 25.0	31.9	33.9	
Income Level (%)			<0.001†
Lowest	16.2	27.1	
Lower Middle	26.3	28.6	
Upper Middle	29.6	20.3	
Highest	28.0	24.0	
Smoking History (%)			0.053
< 5 Packs	59.0	55.6	
≥ 5 Packs	41.0	44.4	
Alcohol Consumption (%)			<0.001†
< 1 Time a Month	43.5	50.1	
1–4 Times a Month	34.5	28.7	
≥ 2 Times a Week	22.0	21.1	
Stress (%)			0.004†
Low	72.5	68.2	
High	27.5	31.8	
Nutritional Factors			
Total Calories (%)	95.76	95.77	0.994
Protein (%)	145.70	134.07	<0.001*
Water (%)	46.22	42.80	0.002*
Sodium (%)	351.73	341.28	0.179
Fat Distribution (%)	17.59	14.87	<0.001*
Carbohydrate Distribution (%)	66.10	69.71	<0.001*

* Significant difference in mean values based on a linear regression analysis with complex sampling, P < 0.05.

^†^ Chi-square test with the Rao-Scott correction and with complex sampling. The level of significance was set to P < 0.05.

An unadjusted analysis revealed statistically significant differences in the protein, water, fat, and carbohydrate intakes between the two groups (P < 0.05 for each). However, the fully adjusted analysis revealed that only the fat intake was significantly different between the two groups (EV of fat intake = -0.57, 95% CI = -1.13 to -0.13, P = 0.045). The fully adjusted analysis found no significant differences in the total calorie intake, protein intake, water intake, sodium intake, and carbohydrate intake between the two groups (Table 2).

**Table 2:** Estimated value (EV) of olfactory dysfunction for each nutritional factor based on simple and multiple linear regression analyses with complex sampling (reference = control group).

Nutritional Factors	Simple Regression			Multiple Regression		
	EV	95% CI	P-value	EV	95% CI	P-value
Total Calories (%)	0.01	-2.67, 2.69	0.994	0.78	-1.95, 3.50	0.575
Protein Intake (%)	-11.63	-16.46, -6.79	< 0.001*	-3.01	-7.86, 1.84	0.224
Water Intake (%)	-3.42	-5.60, -1.24	0.002*	-0.97	-3.15, 1.21	0.381
Na Intake (%)	-10.45	-25.68, 4.79	0.179	-8.72	-23.07, 5.64	0.234
Fat Intake (%)	-2.72	-3.37, -2.08	< 0.001*	-0.57	-1.13, -0.13	0.045*
Carbohydrate Intake (%)	3.61	2.61, 4.61	< 0.001*	0.80	-0.07, 1.66	0.071

* Significant at P < 0.05.

The subgroup analysis according to age and sex revealed that olfactory dysfunction was associated with reduced fat consumption (EV = -2.30, 95% CI = -4.16 to -0.43, P = 0.016) and increased carbohydrate intake (EV = 2.80, 95% CI = 0.55 to 5.05, P = 0.015) among young females. Olfactory dysfunction was also associated with reduced fat intake among middle-aged females (EV = -1.26, 95% CI = -2.37 to -0.16, P = 0.025). Alternatively, among young males, olfactory dysfunction was associated with reduced protein intake (EV = -26.41, 95% CI = -45.14 to -7.69, P = 0.006) (Table 3).

**Table 3:** Subgroup analysis of estimated values (EVs) and 95% confidence intervals (CIs) of olfactory dysfunction for each nutritional factor based on multiple linear regression analysis with complex sampling (reference = control group) according to age and sex.

Nutritional Factors	Age Groups					
	20–39 (y)		40–59 (y)		60+ (y)	
Male	EV (95% CI)	P-value	EV (95% CI)	P-value	EV (95% CI)	P-value
Total Calories (%)	-6.61 (-17.29, 4.07)	0.225	0.23 (-7.37, 7.84)	0.952	0.68 (-4.03, 5.39)	0.776
Protein Intake (%)	-26.41 (-45.14, -7.69)	0.006*	6.31 (-10.06, 22.68)	0.450	-3.95 (-12.13, 4.24)	0.344
Water Intake (%)	-6.57 (-13.92, 0.79)	0.080	-2.21 (-7.86, 3.44)	0.443	-1.05 (-4.70, 2.60)	0.572
Na Intake (%)	-24.02 (-75.67, 27.64)	0.362	22.76 (-27.05, 72.57)	0.370	-25.97 (-55.42, 3.48)	0.084
Fat Intake (%)	-0.36 (-2.43, 1.72)	0.736	0.52 (-1.37, 2.41)	0.590	-0.54 (-1.31, 0.34)	0.232
Carbohydrate Intake (%)	1.83 (-1.66, 5.32)	0.304	-0.71 (-3.54, 2.13)	0.624	0.24 (-1.46, 1.95)	0.781
**Female**						
Total Calories (%)	4.78 (-3.66, 13.17)	0.267	4.21 (-2.02, 10.43)	0.185	1.39 (-2.84, 5.62)	0.520
Protein Intake (%)	3.31 (-10.35, 16.97)	0.634	2.58 (-8.46, 13.62)	0.647	-2.72 (-8.48, 3.03)	0.353
Water Intake (%)	0.51 (-5.11, 6.13)	0.858	2.94 (-3.10, 8.98)	0.340	-0.62 (-3.25, 2.01)	0.644
Na Intake (%)	-5.94 (-39.48, 27.48)	0.728	-16.18 (-42.66, 10.29)	0.231	-0.91 (-25.60, 23.78)	0.942
Fat Intake (%)	-2.30 (-4.16, -0.43)	0.016*	-1.26 (-2.37, -0.16)	0.025*	-0.26 (-1.00, 0.49)	0.495
Carbohydrate Intake (%)	2.80 (0.55, 5.05)	0.015*	1.42 (-0.47, 3.30)	0.140	0.46 (-0.54, 1.46)	0.370

* Significant at P < 0.05.

## Discussion

Olfactory dysfunction was found to be associated with reduced fat intake in this study, and this relationship was particularly evident in the young and middle-aged female groups. This finding is consistent with that obtained in a previous study of elderly women [1]. However, it is unclear why olfactory dysfunction is associated with decreased fat intake. It can be hypothesized that decreased olfactory function could affect food preferences (leading to low fat intake). However, considering the cross-sectional design of this study, the possibility of a reverse causality, i.e., a decreased intake of fat might affect olfactory function, cannot be excluded. In a recent cohort study, higher intakes of n-6 PUFAs, margarine, nuts, and fish showed lower odds ratios for olfactory impairment [8], and the consumption of these types of fat has an anti-inflammatory effect that is mediated by their antioxidant action and their modulation of signal transduction pathways [8, 18]. Insufficient levels of PUFAs are associated with neuropsychiatric diseases, such as major depression, bipolar disorder, schizophrenia, Alzheimer’s disease and attention-deficit hyperactivity disorder [19], which are diseases related to olfactory impairment [20]. Our previous study demonstrated that the consumption of a low-fat diet is associated with hearing loss in an elderly population [21], which suggests that the consumption of a low-fat diet might be related to sensory nerve function. Therefore, decreased fat intake might affect not only hearing but also olfaction.

In this study, olfactory dysfunction was not associated with total calorie intake. However, whether olfactory dysfunction affects total food intake is controversial. Ferris et al. reported that anosmic subjects and healthy controls do not present differences in several anthropometric measures, including BMI [5], or in nutrient intake or eating frequency [5]. According to the results of their study, anosmic subjects do not appear to be at obvious nutritional risk. Duffy et al. also stated that anosmia is not associated with the BMI or relative body fat content (waist-to-hip ratio) [1]. Chauhan J. described that enhanced flavor is not associated with overall energy intake [22]. In contrast, severe chemosensory loss has been associated with low energy intake among advanced cancer patients [23], and individuals with olfactory dysfunction eat less after the onset of olfactory dysfunction [7]. One study reported that change of taste and smell related with weight loss [24]. We think that olfactory dysfunction affects only taste preference, not the homeostasis. It means that decrease of fat intake could be compensated with other carbohydrate diet. Therefore, neither total calories intake nor BMI was different between olfactory dysfunction and control group.

Olfactory dysfunction was not found to be associated with sodium intake. A previous study found that individuals with olfactory dysfunction use more spices and eat more salty foods [1, 7]. In this study, we measured sodium intake (g/ day) using the complete 24-hour recall method. The sodium levels were calculated in reference to the nutrient concentrations in foods according to the Korean Food Composition Table. The perception of and preference for salty flavors may be unrelated to sodium consumption because factors other than taste may influence dietary sodium consumption [25]. Therefore, the preference for sodium and the total sodium intake follow different patterns.

Large population-based studies based on smell tests have shown that women are more sensitive to odor and that olfactory function is superior in women and therefore indicate the existence of gender-related differences in olfactory function [26–28]. Previous studies have found that olfactory loss is more likely to be associated with changes in eating habits in women rather than men [11, 29]. In line with these findings, our study found that younger individuals and females in the olfactory dysfunction group exhibited more significant changes in nutritional intake, particularly increased carbohydrate intake. To the best of our knowledge, no other study has evaluated olfactory dysfunction and the resulting changes in nutritional factors in young adults. One possible explanation for these findings is that younger individuals are more sensitive to olfactory dysfunction and may compensate for their decreased olfaction by seeking a high-carbohydrate diet. In a previous study, olfactory dysfunction was found to be associated with an increased intake of sweets [1].

A decrease in protein consumption among young males with olfactory dysfunction was observed, and a possible explanation for this result has not been well documented. The amino acid composition of dietary proteins contributes to cerebral functions and participates in the elaboration of neurotransmitters [30]. Therefore, decreased protein intake in young males could result in decreased olfactory function. Regarding this issue, there is a need for additional research measuring objective chemosensory function and food-seeking behavior to determine the mechanistic basis for the relationship between food selection and olfactory function.

The present study has several advantages. This study compared the associations between olfactory dysfunction and macronutrient intake among different age groups using a large population-based survey. The sample was weighted by statisticians to better reflect the general population. We adjusted for age, sex, BMI, income, smoking history, alcohol consumption, and stress level, which could act as confounding factors of olfaction [31]. Despite these advantages, this study has several limitations. First, the definition of the study population with olfactory dysfunction is based on a subjective questionnaire using one simple question. However, in clinical practice, few studies have objectively tested olfactory dysfunction. Therefore, few normal ranges of reference are available. Despite this limitation, this study sheds light on self-reported olfactory dysfunction in the general population and its effect on diet. Second, we could not find sufficient explanations for our results. Third, the possibility of residual confounding factors that were not considered in this study cannot be excluded. Finally, the study was subject to the same limitations that affect all cross-sectional studies, including possible reverse causality; therefore, our results should be interpreted with caution.

## Conclusions

Based on an analysis of large population-based survey data, olfactory dysfunction was found to be associated with reduced fat intake among young and middle-aged females, with increased carbohydrate intake among young females, and with reduced protein intake among young males. These data show that loss of olfactory function may differentially affect eating behavior depending on age and sex.

## Supporting Information

S1 Table Proportion of anosmia according to age and sex. (DOCX)
