# Supplementary material for: Olfactory Dysfunction Is Associated with the Intake of Macronutrients in Korean Adults
Source: PLoS One. 2016 Oct 10;11(10):e0164495. doi: 10.1371/journal.pone.0164495 (PMC5056727; doi:10.1371/journal.pone.0164495)
Supplement: S1 Table — (DOCX) [file pone.0164495.s001.docx]

**Supplement 1 Table** Percentage of participants with anosmia according to age and sex

|  | | Total | Control | Olfactory Dysfunction |
| --- | --- | --- | --- | --- |
| Male | | 9,827 | 9,272 (94.4%) | 555 (5.6%) |
|  | 20-39 (y) | 2,689 | 2,618 (97.4%) | 71 (2.6%) |
|  | 40-59 (y) | 3,556 | 3,394 (95.4%) | 162 (4.6%) |
|  | 60+ (y) | 3,582 | 3,260 (91.0%) | 322 (9.0%) |
| Female | | 14,663 | 13,886 (94.7%) | 777 (5.3%) |
|  | 20-39 (y) | 4,428 | 4,326 (7.7%) | 102 (2.3%) |
|  | 40-59 (y) | 5,471 | 5,208 (95.2%) | 263 (4.8%) |
|  | 60+ (y) | 4,764 | 4,352 (91.4%) | 412 (8.6%) |
